# Constitutively decreased *TGFBR1 *allelic expression is a common finding in colorectal cancer and is associated with three *TGFBR1 *SNPs

**DOI:** 10.1186/1756-9966-29-57

**Published:** 2010-05-25

**Authors:** Boris Pasche, Kari B Wisinski, Maureen Sadim, Virginia Kaklamani, Michael J Pennison, Qinghua Zeng, Naresh Bellam, Jacquelyn Zimmerman, Nengjun Yi, Kui Zhang, John Baron, Daniel O Stram, M Geoffrey Hayes

**Affiliations:** 1 Division of Hematology/Oncology, The University of Alabama at Birmingham and UAB Comprehensive Cancer Center, Birmingham, AL 35203, USA; 2 Section of Hematology/Oncology, University of Wisconsin School of Medicine and Public Health, Madison, WI 53792, USA; 3 Cancer Genetics Program, Division of Hematology/Oncology, Department of Medicine, Feinberg School of Medicine, Northwestern University, Chicago, IL 60611, USA; 4 Section of Statistical Genetics, Department of Biostatistics, School of Public Health, The University of Alabama at Birmingham, Birmingham, AL 35203, USA; 5 Departments of Medicine and Community and of Community and Family Medicine, Dartmouth, Hanover, NH 03755, USA; 6 Department of Preventive Medicine, Keck School of Medicine, University of Southern California, Los Angeles, CA 90033, USA; 7 Division of Endocrinology, Metabolism, and Molecular Medicine, Department of Medicine, Feinberg School of Medicine, Northwestern University, Chicago, IL, USA; 8 Center for Genetic Medicine, Feinberg School of Medicine, Northwestern University, Chicago, IL, USA; 9 Department of Anthropology, Northwestern University, Evanston, IL, USA

## Abstract

**Purpose:**

Constitutively decreased *TGFBR1 *allelic expression is emerging as a potent modifier of colorectal cancer risk in mice and humans. This phenotype was first observed in mice, then in lymphoblastoid cell lines from patients with microsatellite stable colorectal tumors.

**Patients and Methods:**

We assessed the frequency of constitutively decreased *TGFBR1 *allelic expression and association with SNPs covering the *TGFBR1 *locus using RNA and DNA extracted from the peripheral blood lymphocytes of 118 consecutive patients with biopsy-proven adenocarcinoma of the colon or the rectum.

**Results:**

We found that 11(9.3%) of 118 patients exhibited decreased *TGFBR1 *allelic expression (*TGFBR1 *ASE). *TGFBR1 *ASE was strongly associated with three SNPs in linkage disequilibrium with each other: rs7034462 (p = 7.2 × 10^-4^), TGFBR1*6A (p = 1.6 × 10^-4^) and rs11568785 (p = 1.4 × 10^-4^).

**Conclusion:**

These results confirm the high prevalence of constitutively decreased *TGFBR1 *allelic expression among patients with colorectal cancer. The association of this phenotype with TGFBR1*6A, rs7034462 and rs1156875 suggests an association between *TGFBR1 *SNPs and colorectal cancer, which warrants additional studies.

## Introduction

Clues regarding important genetic targets in colorectal cancer have come from the study of two hereditary neoplastic syndromes: Familial Adenomatous Polyposis (FAP) and Lynch syndrome, formerly named hereditary non-polyposis colorectal cancer (HNPCC). Although the genetic mechanisms underlying FAP and Lynch syndrome are well-understood, they only account for approximately 0.2% and 2% of all colorectal cancers, respectively. Inherited variants of the *MYH *gene have been shown to cause *MYH*-associated polyposis and are thought to account for an additional 1% of all colorectal cancers. Germline mutations of the *STK11 *gene underlie the Peutz-Jeghers syndrome, and mutations of *SMAD4 *and *BMPR1A *cause juvenile polyposis. Collectively, these syndromes account for 3 to 6% of all colorectal cancers[1].

Much of the remaining familial colorectal cancers and a large proportion of sporadic cases are likely due to low-penetrance mutations, i.e. mutations that have low frequency of association with a specific phenotype[2]. Several recent genome-wide association studies have identified ten additional low penetrance susceptibility alleles including *BMP2*[3], *BMP4*[3] and *SMAD7*[3,4], which all belong to the Transforming Growth Factor Beta (TGF-β) superfamily of growth factors. These findings provide strong support for the notion that the TGF-β signaling pathway is implicated in colorectal cancer susceptibility[5].

We have previously mapped *TGFBR1 *to 9q22[6], and our search for *TGFBR1 *tumor-specific mutations led us to the discovery of a polymorphic allele of the type I receptor, TGFBR1*6A (6A)[6]. This allele has a deletion of three alanines within a 9-alanine stretch of TGFBR1 signal sequence, which results in decreased TGFBR1-mediated signaling[7,8]. The fact that a significantly higher 6A allelic frequency was found among patients with a diagnosis of cancer than among healthy controls prompted us to postulate that 6A may act functionally as a tumor susceptibility allele[6]. Over the past few years, some studies have confirmed an association between 6A and cancer, but others have failed to establish any correlation. A combined analysis of 17 case control studies that included more than 13,000 cases and controls showed that 6A allelic frequency was 44% higher among all cancer cases (0.082) than among controls (0.057) (p < 0.0001)[9]. The first combined analysis of the six studies assessing 6A in colon cancer cases and controls indicated that 6A carriers are at increased risk of developing colorectal cancer (O.R. 1.20, 95% CI 1.01-1.43)[10], but a large case control study performed in Sweden did not confirm this association (O.R. 1.13, 95% CI 0.98-1.30)[11].

To test the hypothesis that constitutively decreased TGFBR1 signaling modifies colorectal cancer risk, we developed a novel mouse model of *Tgfbr1 *haploinsufficiency[12]. We crossed *Tgfbr1 *haploinsufficient mice with *Apc*^*Min*/+ ^mice, one of the most commonly used models of colorectal cancer, and found that constitutively decreased Tgfbr1 signaling is a potent modifier of colorectal cancer risk[12]. These animal findings prompted validation in patients with colorectal cancer. We chose patients with microsatellite instability (MSI) negative colorectal cancer in order to exclude most patients with somatically acquired *TGFBR2 *mutations, a common finding in MSI-positive colorectal cancer[13]. This led to the identification of two novel haplotypes associated with decreased *TGFBR1 *allelic expression and markedly increased risk of colorectal cancer[14].

A recent report suggests that the *TGFBR1 *ASE phenotype is non-existent in patients with sporadic colorectal cancer[15]. We undertook this study to assess whether this is indeed the case, and to establish the frequency of this novel phenotype in unselected, consecutively recruited patients with colorectal cancer. The second goal of this study was to determine the association of constitutively decreased *TGFBR1 *allelic expression with haplotype tagging SNPs at the *TGFBR1 *locus. Our findings confirm our original discovery of a high frequency of constitutively decreased TGFBR1 allelic expression in patients with colorectal cancer. They further establish its association with TGFBR1*6A as well as two additional haplotype tagging SNPs.

## Methods

### Patients

The series of colorectal cancer cases from Northwestern University Medical and Surgical Clinics in Chicago have been previously described [16]. They were enrolled as part of IRB-approved protocols. Briefly, consecutive cases with a biopsy-confirmed diagnosis of colorectal adenocarcinoma were recruited from the medical and surgical oncology clinics affiliated with the Northwestern Medical Faculty Foundation and U.S. Oncology during the years 2000 and 2006. RNA was only available for 118 of the 199 colorectal cases because of either a shortage of blood RNA kits during part of the study or poor quality of the extracted RNA.

### DNA/RNA extraction and cDNA synthesis

DNA was extracted from whole blood samples using the QIAamp DNA Blood Mini Kit (Qiagen, Valencia, CA) and was stored at -20°C until use for genotyping. RNA was extracted from whole blood samples using the Paxgene Blood RNA Kit (Qiagen, Valencia, CA) prior to reverse transcription with Taqman^® ^Reverse Transcription Reagents (Applied Biosystems, Foster City, CA).

### Assessment of constitutively decreased *TGFBR1 *allelic expression

We used the methods described in our recent report identifying constitutively decreased *TGFBR1 *allelic expression in humans[14]. Briefly, germline DNA from all patients with available DNA and RNA was genotyped for the following four 3'-UTR SNPs: rs334348, rs334349, rs1590 and rs7871490. The ratio of the two alleles in the cDNA of the transcript was normalized with the ratio of the two alleles in genomic DNA, applying the following formula: cDNA (peak area common allele/peak area rare allele) divided by gDNA (peak area common allele/peak area rare allele). Each SNP was assayed with two independent cDNA preparations, each in duplicate so that the ASE was calculated as the average of 4 different ratios. The diagnostic criteria for the *TGFBR1 *ASE phenotype were the same as in our prior report, i.e. a ratio of cDNA/gDNA either ≥ 1.5 or ≤ 0.67[14].

### Selection of SNPs

Using phase II HapMap data for the HapMap European (CEU) sample for *TGFBR1*, we selected 18 tag SNPs in addition to TGFBR1*6A and genotyped the 19 variants in all colorectal cancer cases. The tag SNPs were designed to give pairwise r^2 ^> 0.8 for all common SNPs in the *TGFBR1 *region. A check using release 22 (April 2007) of the HapMap Phase II data showed that this pairwise r^2 ^value was achieved for 57 of 58 common SNPs identified in HapMap Phase II. The remaining common SNP was tagged successfully (r^2 ^> 0.8) using a haplotype of two of the tag SNPs. The mean r^2 ^for the 58 SNPs was 0.967 indicating excellent coverage of this region with our 18 tag SNPs.

### Statistical analyses

We used standard chi-square tests to assess the significance of allele frequency differences between ASE individuals (>1.5 or <0.67; N = 11) and the remainder of the cohort.

## Results

### Frequency of the *TGFBR1 *ASE phenotype

In this cross sectional study of 118 consecutively-recruited patients with colorectal cancer 74 (62.7%) individuals were heterozygous for informative *TGFBR1 *SNPs. Eleven (9.3%) patients had evidence of constitutively decreased *TGFBR1 *allelic expression, i.e. a ratio of cDNA/gDNA either ≥ 1.5 or ≤ 0.67[14]. Median age at diagnosis was 60 years in subjects with *TGFBR1 *ASE and in those without and the sex distribution was similar as well (Table 1). The frequency of constitutively decreased *TGFBR1 *allelic expression among Caucasian patients was 10.2% (10/98)and 7.1% (1/14) in the African-American population. None of the patients with self-described Hispanic (3) or Asian (3) ethnicity had decreased *TGFBR1 *allelic expression. Fifty-five percent of the patients with decreased *TGFBR1 *allelic expression had a primary colon cancer. This was similar to the 66% with primary colon cancer in patients with normal *TGFBR1 *allelic expression (p = 0.507; Fisher's Exact Test). The stage at diagnosis was equivalent in both groups with only 9% presenting with stage I disease and 27% of those with normal *TGFBR1 *allelic expression having stage IV disease, similar to the 36% in those patients with decreased *TGFBR1 *allelic expression (p = 0.498; Fisher's Exact Test). A family history of colorectal cancer in a first or second degree relative was present in 29% of all patients and was comparable between the two groups (Table 1).

**Table 1:** Demographics and clinical characteristics of patients with and without constitutively decreased *TGFBR1 *allelic expression (*TGFBR1 *ASE).

	All patients		*TGFBR1 *ASE +		*TGFBR1 *ASE -	
Age, years	No	%	No	%	No	%
Median age	59.5		64.0		59	
Range	35-84		52-77		35-84	
						
Sex						
Female	55	46.6	4	3.4	51	43.2
Male	63	53.4	7	5.9	56	47.5
						
Tumor location						
Colon	77	65.3	6	5.1	71	60.2
Rectum	40	33.9	5	4.2	35	29.7
Both	1	0.8	0	0	1	0.8
						
Ethnic status						
Caucasian	98	83.1	10	8.5	88	7.5
African American	14	11.9	1	0.8	13	11.0
Asian	3	2.5	0	0	3	2.5
Hispanic	3	2.5	0	0	3	2.5
						
Stage at diagnosis						
Stage 1	11	9.3	1	0.8	10	8.5
Stage 2	30	25.4	5	4.2	25	21.2
Stage 3	44	37.3	1	0.8	43	36.4
Stage 4	33	28.0	4	3.4	29	24.6
						
Family history						
No	76	64.4	7	5.9	69	58.5
Yes	34	28.8	3	2.5	31	26.3
Unknown	8	6.8	1	0.8	7	5.9

### Association of *TGFBR1 *SNPs with *TGFBR1 *allele-specific expression

Three SNPs in linkage disequilibrium with each other were strongly associated with *TGFBR1 *ASE: rs7034462 (p = 7.2 × 10^-4^), TGFBR1*6A (p = 1.6 × 10^-4^) and rs11568785 (p = 1.4 × 10^-4^) (Table 2). TGFBR1*6A is located within the coding sequence of exon 1 and the other two SNPs are located within introns. rs7034462 is located 9.2 kb upstream of exonn 1 and rs11568785 is located 850 bp downstream of exonn 5 and 1.18 kb upstream of exonn 6. These results are consistent with our earlier findings as each of these SNPs was significantly associated with *TGFBR1 *ASE in our original study. For example, in this study six (54.5%) of the 11 patients with TGFBR1 ASE carried the TGFBR1*6A allele. In our previous report 14 (48.3%) of the 29 patients with TGFBR1 ASE carried the TGFBR1*6A allele. This provides additional evidence of a central role for TGFBR1*6A in colorectal cancer, especially as it relates to the *TGFBR1 *ASE phenotype. Studies are currently in progress to validate the association of *TGFBR1 *SNPs with colorectal cancer risk.

**Table 2:** Association of TGFBR1 SNPs with constitutively decreased *TGFBR1 *allelic expression (*TGFBR1 *ASE).

	Frequency Allele 2			
**SNP**	**ASE < 0.67 or > 1.5**	**1.5 > ASE > 0.67**	**P**	**OR**

rs4742761	0.14	0.25	0.38	0.5

rs2416666	0.19	0.19	0.98	1.0

rs7874183	0.13	0.28	0.20	0.4

rs7034462	0.31	0.05	7.2 × 10-4	8.3

rs10819634	0.06	0.26	0.08	0.2

rs1888223	0.50	0.30	0.11	2.3

9A/6A	0.31	0.04	1.6 × 10-4	10.9

rs10988705	0.00	0.04	0.42	n/a

rs6478974	0.50	0.47	0.82	1.1

rs10739778	0.38	0.36	0.89	1.1

rs2026811	0.25	0.32	0.57	0.7

rs10512263	0.00	0.11	0.16	n/a

rs11568785	0.25	0.02	1.4 × 10-4	16.0

rs334348	0.31	0.39	0.55	0.7

rs7871490	0.50	0.46	0.77	1.2

rs334349	0.25	0.43	0.19	0.5

rs7850895	0.07	0.06	0.87	1.2

rs1590	0.25	0.39	0.28	0.5

rs1626340	0.25	0.32	0.57	0.7

## Discussion

These findings confirm the relatively high frequency of the *TGFBR1 *ASE phenotype in patients with colorectal cancer. The phenotype frequency among Caucasian patients included in this study (10.2%) is similar to that of the Caucasian patients studied in our earlier report (12.0%)[14]. Intriguingly, Guda et al. did not identify any individual with the *TGFBR1 *ASE phenotype among 96 patients with colorectal cancer without any family history of colorectal cancer[15]. They identified a low (1.9%) frequency of the phenotype among 102 patients with familial colon neoplasia[15]. Our patient population was not entirely similar to the patients with sporadic colorectal cancer reported in the Guda et al. study. The average age at diagnosis of the patients with sporadic colorectal cancer in that study was 69 while the median age at diagnosis in our study was 60. Also, the proportion of Caucasians was more than 10% lower in the Guda study (71%) than in our study (83%).

However, the main difference between these two studies is the tissue from which DNA and RNA were extracted. In both our original report[14] and in this study we used lymphoblastoid cell lines and peripheral blood lymphocytes whereas Guda et al. extracted DNA and RNA from the normal-appearing mucosa layer of the colon from patients with sporadic colorectal cancer[15]. The authors assumed that if TGFBR1 ASE were a driver of colorectal cancer, the lower expression of one *TGFBR1 *allele should likely also be evidenced in colon epithelial cells from affected individuals.

While we agree with this reasoning, it possible that the TGFBR1 allelic expression ratio in lymphoblastoid cell lines is not the same as in normal appearing colonic epithelium. We have previously shown that TGFBR1*6A, one of the SNPs previously associated with the *TGFBR1 *ASE phenotype[14], is somatically acquired in the normal appearing colonic epithelium of a small proportion of patients with colorectal cancer[17]. This provides support for the notion that either somatically-acquired mutations or epigenetic changes may affect the *TGFBR1 *gene in the normal appearing colonic epithelium and may therefore affect determination of the *TGFBR1 *ASE phenotype. Several recent studies have demonstrated that genetic alterations within the stroma may have a potent effect on cancer progression[18]. Hence, another potential explanation for these differences is altered stromal TGF-β signaling, which is emerging as a potent modifier of cancer susceptibility[19]. Identification of the *TGFBR1 *ASE phenotype in African American patients suggests that this phenotype may not be exclusively found in Caucasians. Additional studies in various ethnic groups are warranted.

In summary our results confirm the high frequency of the TGFBR1 ASE phenotype among patients with colorectal cancer and suggest a central role of the *TGFBR1 *locus in the etiology of this disease.

### Competing interests

Boris Pasche has filed patents related to the role of constitutively decreased TGFBR1 expression as it relates to cancer risk.

### Authors' contributions

BP: Conception and design. KBW, VK: Provision of study material or patients. KBW: Collection and assembly of data. NY, KZ, DOS, MGH: Data analysis and interpretation. BP: Manuscript writing. BP, KBW, MS, VK, MP, QZ, NB, JZ, NY, KZ, JB, DOS, MGH: Final approval of manuscript.

### Funding

Supported by grants from the UAB startup funds and grants CA112520, CA108741, CA137000 and 5P60AR048098 from the NIH.

### Presented in part

Abstract # 95, American Association for Cancer Research 100th Annual Meeting 2009 in Denver, CO
